# Antidepressant indatraline induces autophagy and inhibits restenosis via suppression of mTOR/S6 kinase signaling pathway

**DOI:** 10.1038/srep34655

**Published:** 2016-10-03

**Authors:** Yoon Sun Cho, Chih-na Yen, Joong Sup Shim, Dong Hoon Kang, Sang Won Kang, Jun O. Liu, Ho Jeong Kwon

**Affiliations:** 1 Chemical Genomics Global Research Laboratory, Department of Biotechnology, Translational Research Center for Protein Function Control, College of Life Science & Biotechnology, Yonsei University, Seoul 120-749, Republic of Korea; 2 Department of Pharmacology and Molecular Sciences, Johns Hopkins School of Medicine, 725 N. Wolfe St, Baltimore, MD 21205, USA; 3 Faculty of Health Sciences, University of Macau, Taipa, Macau 999078, China; 4 Division of Life and Pharmaceutical Sciences, Ewha Womans University, Seoul 120-750, Republic of Korea; 5 Department of Internal Medicine, Yonsei University College of Medicine, Seoul 120-752, Republic of Korea

## Abstract

Indatraline is an antidepressive agent and a non-selective monoamine transporter inhibitor that blocks the reuptake of neurotransmitters (dopamine, serotonin, and norepinephrine). In this study, we report that indatraline induces autophagy via the suppression of mTOR/S6 kinase signaling. Autophagy induction was examined by a cell-based high content screening system using LysoTracker, which was followed by monodansylcadaverine staining and transmission electron microscope observation. Indatraline increased the number of EGFP-LC3 cells expressing autophagosomes in the cytoplasm. Conversion of LC3 was further validated by immunoblotting. Indatraline induced autophagy by affecting the AMPK/mTOR/S6K signaling axis and had no influence on the PI3K/AKT/ERK signaling. Moreover, indatraline induced autophagy in smooth muscle cells (SMCs); further, it exhibited therapeutic potential for restenosis by inhibiting SMC accumulation in a rat restenosis model. These results provide new insights into the role of monoamine transporters in autophagy regulation and identify indatraline as a novel agent for inducing autophagy.

Autophagy is an important cellular catabolic process, in which the cellular components are degraded and recycled as nutrients and energy sources. During the life span of a cell, malfunctioning organelles and long-lived proteins are processed by autophagy. When autophagy is activated, the membrane structure of the autophagosome is formed by the atg5-atg12 complex and LC3 recruitment. The cytosolic form of LC3 (LC3-I) is cleaved into the membrane-bound form (LC3-II), and the membrane matures into an autophagosome^1^,^2^,^3^. The autophagosome then fuses with the lysosome, resulting in lysosomal degradation of the cellular components. Autophagy is known to regulate cell survival via the flux of sequential events, although the specific underlying mechanism is largely unclear. Autophagy-induced cell death is classified as type II cell death, which is distinct from caspase-dependent apoptosis^4^.

Autophagy has been implicated in various diseases such as atherosclerosis, restenosis, neurodegenerative diseases, and cancer^5^,^6^,^7^,^8^. Atherosclerosis is a long-term inflammatory disease of the arterial wall that is primarily caused by plaque destabilization and rupture^9^. It can be treated by angioplasty or stent placement to relieve the blockage^10^. In atherosclerosis, macrophages are responsible for atherosclerotic plaque destabilization, and therefore, selective induction of macrophage death is a desirable method for removing atherosclerotic plaques^11^. Sirolimus- and everolimus (rapamycin derivatives)-eluting stents are currently in clinical use for this effect in atherosclerosis treatment^8^,^12^. However, atherosclerosis treatment can be followed by restenosis, which occurs when new tissue grows inside the stent, and scar tissues grow from underneath the new healthy tissues. Restenosis is currently treated with repeat angioplasty, bypass surgery, or intravascular radiation to prevent reoccurrence. Therefore, restenosis-targeting therapies or drugs are urgently needed. In addition to its role in atherosclerosis, autophagy may also provide a possible mechanism for degrading accumulated fibrils and amyloid plaques in Parkinson’s or Alzheimer’s disease. Autophagy has also been implicated in various stages of cancer^13^. In apoptosis-deficient cancer cells, autophagy can be induced to promote cell death; on the other hand, in growing tumor cells, autophagy can be used to maintain survival until angiogenesis provides oxygen and nutrients. In this case, autophagy should be inhibited to suppress the survival of tumor cells^14^. Some autophagy inducers promote autophagy and apoptosis simultaneously, which leads to synergistic or additive effects on cell death^15^.

An increasing number of reports on the pathological roles of autophagy in human diseases suggest that autophagy inducers can be potentially utilized as drugs^16^. Notably, rapamycin^17^ is an effective autophagy inducer that is currently in clinical use for treatment of atherosclerosis^8^,^18^ and other diseases. Recently, autophagonizer, a new synthetic small molecule, was discovered via phenotypic cell-based screening; however, its underlying mechanism was different from that of rapamycin^19^,^20^. Autophagonizer did not affect mTOR signaling, which suggests the presence of other small molecules that induce autophagy, albeit with unspecified mechanisms that will be new pathways for exploring autophagy-related biology and chemotherapeutic development.

In our effort to find new small molecules with autophagic activities, indatraline [(1*R*, 3S)-3-(3, 4-dichlorophenyl)-*N*-methyl-2,3-dihydro-1*H*-inden-1-amine, Lu 19-005] was identified as an autophagy inducer from the Johns Hopkins Drug Library (JHDL), which consists of 2,386 clinical drugs^21^,^22^. Indatraline is a non-selective monoamine transporter inhibitor that blocks the reuptake of dopamine, norepinephrine, and serotonin with efficacy similar to cocaine, which is a psychostimulant; however, with regard to Indatraline, these effects have been demonstrated to have slower onset and longer duration than those of cocaine, suggesting that the compound may be used to treat cocaine addiction^23^. Indatraline has been used to block the action of methamphetamine and MDMA (3,4-methylenedioxy-N-methylamphetamine) as an antidepressant^24^. However, there has been no report on autophagy induction by indatraline.

We showed that indatraline induced autophagy while simultaneously inhibiting cell proliferation; additionally, it promoted apoptosis-independent cell death leading to effective suppression of restenosis in a rat restenosis model. Thus, this study provides a new chemical probe to reveal the potential relationship between monoamine transporters and autophagy. It may also serve to direct the development of new drugs for treating autophagy-related diseases such as atherosclerosis or restenosis^25^.

## Results

### Indatraline induces autophagy

**Figure 1:**
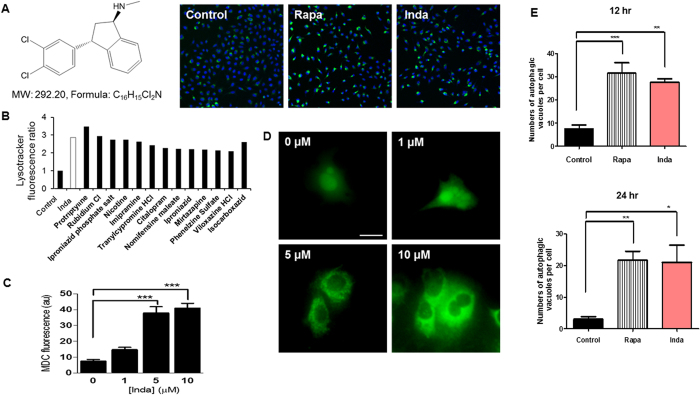
Indatraline induces autophagy in cells. (**A**) Indatraline structure and representative images from HCS analysis. The images show the pseudo color of LysoTracker Red fluorescence. (Control: control DMSO, Rapa: rapamycin, Inda: indatraline). (**B**) High content screening analysis of antidepressants with 2-fold or greater autophagy activity compared to that in the control. (**C**) Quantitative fluorescence measurements of monodansylcadaverine (MDC) staining in indatraline-treated cells (mean ± SEM, ‘au’ represents arbitrary unit, ***indicates p < 0.0001). (**D**) The EGFP-LC3 stable cell line also exhibited an increased number of cells containing vacuoles in the cytoplasm after indatraline treatment. Scale bars represent 20 μm. Images taken from fluorescence microscopy. (**E**) Quantitative analysis of autophagic vacuoles in rapamycin and indatraline treated cells after 12 and 24 h. Bar graph represents number of autophagic vacuoles per cell (mean ± SEM, n = 4, *indicates p < 0.05, **indicates p < 0.005, and ***indicates p < 0.0001).

To identify new compounds that induce autophagy, a clinical drug library, JHDL, was screened with high content screening (HCS). After exposing cells to the clinical drugs in 96-well plates, autophagy induction was detected and quantified by LysoTracker Red staining followed by HCS analysis. LysoTracker Red is a fluorescent dye that stains acidic vacuoles within the cells, increasing fluorescence in the cytoplasm when autophagy is activated. From this chemical screening assay, 220 compounds were found to induce autophagy by at least 2-fold compared with the control induction at a final concentration of 10 μM. Among them, 14 compounds were antidepressants, and indatraline (Fig. 1A) induced autophagy by 2.87-fold compared with the control induction at 10 μM, which was similar to the effects of rapamycin. The 14 antidepressants comprised dopamine, serotonin, norepinephrine, monoamine oxidase, or combinations of two neurotransmitter inhibitors (Fig. 1B). However, indatraline was the only nonspecific neurotransmitter transporter inhibitor that inhibits dopamine, serotonin, and norepinephrine reuptake. Accordingly, it was selected for further investigation.

Autophagy induction by indatraline was further examined by monodansylcadaverine (MDC) staining. MDC is a marker that labels all acidic organelles including lysosomes, which could be used to preliminarily screen autophagy induction^26^. MDC staining after indatraline treatment revealed increased fluorescence at indatraline concentrations as low as 1 μM, similar to the LysoTracker staining result (Fig. 1C; Supplementary Fig. 2). To detect the direct components of autophagy, indatraline-induced autophagy was validated using the COS-7 EGFP-LC3 stable cell line. On induction of autophagy, LC3—a microtubule-associated light chain 3 protein—is converted from its cytosolic form into its membrane-bound form. After indatraline treatment for 24 h, small vacuoles were examined, and EGFP-LC3 fluorescent vacuoles increased concentration-dependently in the cytoplasm (Fig. 1D). The structures of autophagosomes were further examined with TEM images. HeLa cells were treated with 5 μM indatraline or 10 μM rapamycin for 12 and 24 h, respectively. Beginning from 12 h, autophagic vacuoles were clearly detected in indatraline-treated cells (Supplementary Fig. 3). Multi-vesicular bodies and double membrane structures significantly increased in number and size (Fig. 1E).

**Figure 2:**
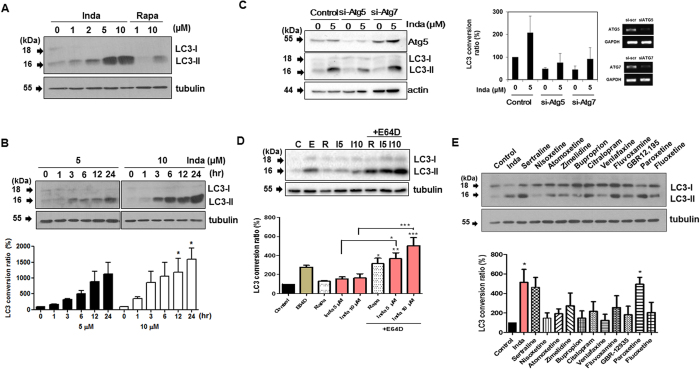
Indatraline-induced autophagy was validated by LC3 conversion immunoblotting. (**A**) LC3 conversion immunoblotting in indatraline-treated HeLa cells. Rapamycin (Rapa) was used as a positive control. All immunoblots detect endogenous LC3 unless specified otherwise. (**B**) Time-course analysis of LC3 conversion. Graph shows the quantitative analysis of LC3 conversion from three independent trials (mean ± SEM, *p < 0.05). (**C**) Knockdown (KD) of ATG5 and ATG7 by siRNA transfection. Indatraline-induced autophagy was inhibited by KD of indicated genes. (Quantification from three independent experiments, mean ± SD.) (**D**) Assessment of autophagic flux in the absence and presence of protease inhibitor, E64D. Cells were incubated with E64D for 1 h prior to indatraline and rapamycin treatment (C: DMSO control, E: E64D, R: rapamycin, I5: indatraline 5 μM, I10: indatraline 10 μM). Quantitative analysis from three independent trials (mean ± SEM, *p < 0.05). (**E**) LC3 conversion induced by different monoamine transporter inhibitors. Inda represents indatraline. Quantitative analysis from three independent trials (mean ± SEM, *p < 0.05).

LC3 conversion resulting from indatraline treatment was further confirmed with LC3 immunoblotting. The concentration-dependent conversion of LC3 was examined in cells treated with indatraline for 24 h (Fig. 2A). LC3 conversion was examined starting from 5 μM in cells. At 5 and 10 μM, indatraline induced strong LC3 conversion beginning at 3 h (Fig. 2B). The knockdown of two of the essential autophagy related proteins, ATG5 and ATG7, abrogated indatraline-mediated autophagy (Fig. 2C). Additionally, the effect of indatraline on autophagic flux was assessed by using E64D, a lysosomal inhibitor. In the LC3 turnover assay, the LC3 conversion ratio of cells treated with E64D alone was compared to cells treated with indatraline alone and cells co-treated with indatraline and E64D^27^. Differences of LC3-II level between cells treated with indatraline alone and cells co-treated with indatraline plus E64D should not be evident if autophagic flux is blocked by indatraline^28^. In our analysis, accumulation of LC3-II was detected in the presence of E64D together with indatraline, indicating the occurrence of autophagic flux upon treatment with indatraline. Cells treated with 5 μM and 10 μM of indatraline in the presence of E64D showed increases of 2-fold and 3-fold, respectively, compared to cells treated with indatraline alone (Fig. 2D). Accordingly, our results demonstrate that indatraline leads to an increase in autophagic flux.

Furthermore, several other antidepressants were tested along with indatraline to validate the involvement of monoamine reuptake in autophagy (Fig. 2E). The serotonin selective monoamine transporter inhibitors sertraline, zimelidine, citalopram, fluvoxamine, and paroxetine induced autophagy by more than 2-fold compared to the dimethyl sulfoxide (DMSO) control. Clearly, autophagy is induced by indatraline, as well as other antidepressants, which indicates that indatraline can be potentially used as a chemical probe for exploring the role of monoamine reuptake in autophagy and its related biological phenotypes. Moreover, the relationship between indatraline-induced cell death and apoptosis was examined by inhibiting caspase activity, which is the hallmark for apoptosis. Indatraline-induced cell death was resistant to z-VAD-fmk treatment, whereas camptothecin-induced cell death was not (Supplementary Fig. 1A). Furthermore, caspase-3 was cleaved only in camptothecin-treated cells (Supplementary Fig. 1B). Collectively, indatraline inhibited cell proliferation and induced apoptosis-independent cell death.

### Indatraline affects AMPK/mTOR/S6K signaling axis

**Figure 3:**
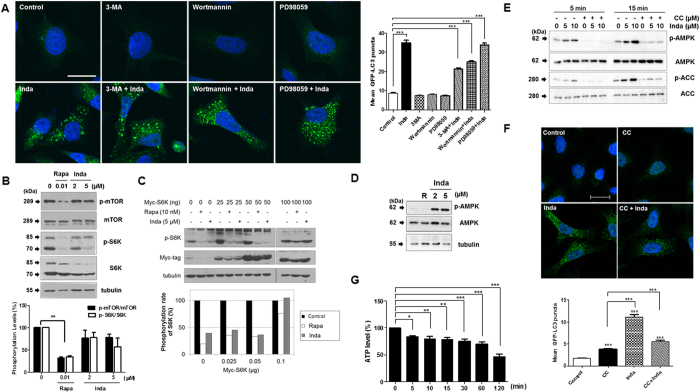
Indatraline suppresses mTOR and S6K phosphorylation. (**A**) LC3 positive autophagosomes were increased by indatraline in HeLa cells (representative images from three independent experiments). Autophagy inhibitors with different modes of action were treated 1 h prior to indatraline. Images are from confocal microscopy. Graph shows quantitative analysis of LC dots from three independent experiments. (**B**) Similar to positive control (Rapa), indatraline decreased the level of phosphorylation of mTOR and its downstream target, i.e., S6K. Graphs are quantifications from three independent experiments (mean ± SEM, **p < 0.005). (**C**) Myc-S6K was transfected into cells. The phosphorylation level of S6K was suppressed by rapamycin and indatraline; however, S6K suppression by indatraline was rescued by S6K overexpression. (**D**) Indatraline evidently increased AMPK phosphorylation. (**E**) Indatraline-induced AMPK activity was inhibited by the co-treatment of compound C (CC). (**F**) LC3-positive autophagosomes were decreased when cells were co-treated with indatraline and CC. Images are representative images from three independent experiments. Quantitative analysis of LC3 vacuoles from three independent experiments (mean ± SEM, ***indicates p < 0.0001). (**G**) ATP level was reduced by indatraline treatment (min). Quantitative analysis from four independent experiments (mean ± SEM compared to control, *indicates p < 0.05, **indicates p < 0.005, and ***indicates p < 0.0001).

To investigate which signaling pathway is involved in indatraline-induced autophagy, cells were pretreated with autophagy inhibitors with different mechanisms of action 1 h prior to 10 μM indatraline and 15 μM rapamycin treatment; 3-MA (10 mM)^29^ and PD98059 (10 μM)^30^ are inhibitors of autophagy that inhibit the PI3K/AKT and MEK/ERK signaling pathways, respectively. As expected, punctate LC3-positive autophagosomes were detected in the cytosol as a result of indatraline and rapamycin treatment for 24 h (Supplementary Fig. 4). Indatraline-induced LC3-positive vacuoles remained in the cytosol regardless of the presence of other autophagy inhibitors (Fig. 3A). However, rapamycin-induced LC3 vacuoles decreased with PI3K/AKT and ERK signal inhibitors (Supplementary Fig. 4). Thus, these results demonstrate that PI3K/AKT and ERK signaling axes are not involved in indatraline-induced autophagy.

Thereafter, the effect of indatraline on the mTOR signaling pathway was examined. Indatraline inhibited mTOR/S6K signaling similar to the action of rapamycin, although with weaker effect (Fig. 3B). However, the pattern was similar to rapamycin in that p-S6K levels were recovered when S6K was overexpressed in cells dose-dependently (Fig. 3C). Since indatraline affects mTOR signaling similar to rapamycin, the upstream signaling of mTOR excluding PI3K/AKT or ERK was further examined with AMP kinase (AMPK). Notably, indatraline activates AMPK (Fig. 3D); however, its effect on AMPK was inhibited by pretreatment with an AMPK inhibitor, i.e., compound C (Fig. 3E). Additionally, the majority of LC3-positive puncta in the cytosol decreased when cells were co-treated with compound C and indatraline (Fig. 3F). AMPK is activated when the AMP/ATP ratio is high, and its activation is one of the essential factors in autophagy induction. Indatraline also decreased intracellular ATP level (Fig. 3G); this indicated that indatraline-induced AMPK activation might be the result of reduced ATP level. Collectively, these data suggest that indatraline triggers robust autophagy via AMPK/mTOR/S6K signaling transduction.

### Indatraline is efficacious in an animal model of restenosis

**Figure 4:**
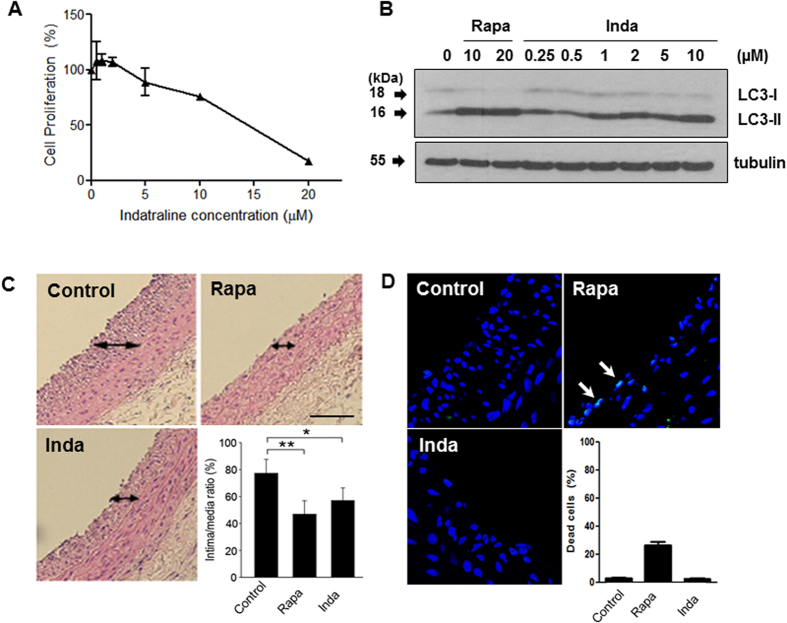
Effect of indatraline on smooth muscle cell (SMC) proliferation and restenosis. (**A**) MTT [3-(4,5-dimethylthiazol-2-yl)-2,5-diphenyltetrazolium bromide] assay with indatraline-treated SMCs. (**B**) LC3 conversion immunoblotting using SMCs. (**C**) Rat carotid artery restenosis model. Indatraline inhibited the accumulation of neointimal SMCs. The arrow indicates the neointimal accumulation of SMCs. The bar graph presents the percentage of neointimal plaque area versus the medial layer area (mean ± standard error of the mean (SEM), n = 4, *p < 0.05, **p < 0.001). (**D**) TUNEL staining of the rat restenosis model. Arrows indicate apoptotic cells. Bar graph presents the percentage of dead cells (mean ± SEM, n = 4; Control: DMSO control, Inda: indatraline, Rapa: rapamycin).

Autophagy induction has therapeutic potential for restenosis, the narrowing of blood vessels caused by the rapid proliferation of smooth muscle cells (SMCs) or macrophages^31^. Restenosis is a chronic inflammatory response, and treatment involves angioplasty, stent, or radiotherapy. Stents eluting rapamycin and its derivatives are used for atherosclerosis treatment. To test indatraline for its potential to treat restenosis, effects of indatraline on cell proliferation and autophagy activity were performed in SMCs. Indatraline inhibited SMC proliferation with an IC_50_ of 15 μM (Fig. 4A). LC3 conversion was validated in both indatraline- and rapamycin-treated SMCs (Fig. 4B). Indatraline induced LC3 conversion at concentrations as low as 1 μM in SMCs. We then tested indatraline in a rat restenosis model. After inducing a balloon injury in the rat carotid artery, 2 μM indatraline was injected. Both rapamycin and indatraline inhibited neointimal accumulation of SMCs (Fig. 4C). Notably, however, restenosis inhibition by indatraline did not involve apoptosis as shown by TUNEL staining, a marker of apoptotic DNA fragmentation (Fig. 4D). Both control and indatraline-treated samples did not exhibit TUNEL-positive cells, although rapamycin-treated samples showed less than 30% of TUNEL-positive cells.

## Discussion

Indatraline is a monoamine transporter inhibitor with antidepressive activity. The present study revealed that the growth-inhibitory activity of indatraline is mediated through autophagy, a form of apoptosis-independent cell death. Monoamine transporter inhibitors and their relationship to autophagy are currently under active investigation. Autophagy inductions by various types of monoamine reuptake inhibitors have been reported. For instance, imipramine, citalopram, and nomifensine are a serotonin-norepinephrine reuptake inhibitor, selective serotonin reuptake inhibitor, and norepinephrine-dopamine reuptake inhibitor, respectively. The mechanisms behind autophagy induction by monoamine reuptake inhibitors have not been clearly identified; however, as autophagy-controlling compounds have the potential to be developed into pharmaceutical drugs, similar to rapamycin, much research in repositioning these drugs has been reported^32^,^33^,^34^,^35^,^36^,^37^,^38^,^39^. In this study, indatraline was identified as a new autophagy inducer from the JHDL, a pharmacologically diverse pool of compounds. Prolonged treatment (72 h) with indatraline decreased cell survival as indicated by a cell proliferation assay, while shorter treatment (for 24 h) was not observed to be toxic during autophagy screening.

**Figure 5:**
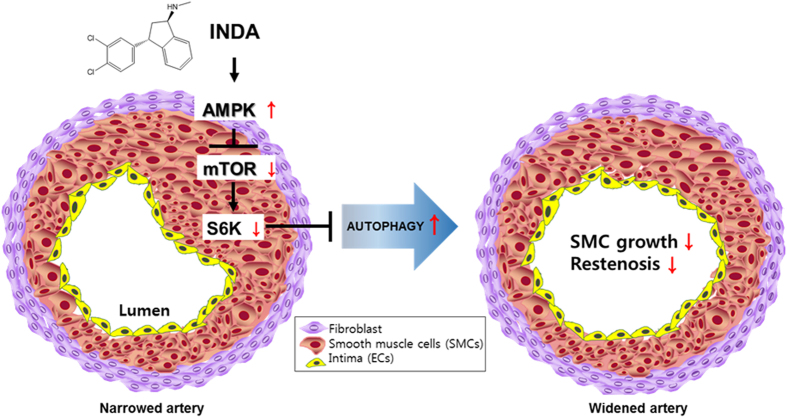
Proposed mechanism of indatraline-induced autophagy. Indatraline induces AMPK activation, followed by the inhibition of mTOR/S6K signaling. This leads to robust autophagy induction. Indatraline effect on autophagy (red arrows). Indatraline-induced autophagy resulted in cell growth inhibition of smooth muscle cells and restenosis in a rat restenosis model. This highlights that indatraline may be a potential restenosis inhibitor through its mechanism of autophagy induction.

As described, autophagy has been implicated in numerous human diseases. These reports also suggest that autophagy inducers have the potential to become therapeutic drugs for atherosclerosis. Rapamycin derivative-eluting stents are currently used for atherosclerosis treatment, as they inhibit the proliferation of macrophages and plaque accumulation. Similarly, as indatraline effectively inhibits SMC proliferation, it could be applied to the therapeutic approach for atherosclerosis with further testing on macrophage and plaque accumulation. Indeed, indatraline was clearly effective in inhibiting restenosis in a rat restenosis model (Fig. 5).

According to our results, indatraline induces autophagy with intracellular signaling that involves the AMPK/mTOR/S6K signaling axis. Prolonged treatment (72 h) of indatraline decreased cell survival from apoptosis-independent cell death. Indatraline revealed similar effects in autophagy signaling when compared to rapamycin. The PI3K signaling pathway is related to autophagy by acting upstream of mTOR, and indatraline inhibited mTOR signaling. Notably, however, the upstream signal did not involve PI3K or AKT, and only AMPK was affected by indatraline. AMPK activity is reported to induce autophagy in cells, which is followed by mTOR inhibition. Likewise, indatraline induced AMPK activity and inhibited mTOR/S6K activity to induce autophagy. Indatraline increased LC3-II accumulation and autophagic flux. Furthermore, some organelles appeared enlarged from indatraline treatment according to the TEM images. These results are similar to LC3-associated phagocytosis (LAP)-like autophagy, which involves water influx into lysosomes by chloroquine^40^. However, chloroquine is conventionally used to inhibit autophagic flux, which differs from indatraline. The precise mechanism by which indatraline induces autophagy should be studied in further detail to understand the effect of indatraline on mTOR/S6K signaling and subsequent increase in autophagy flux with LAP-like phenomenon. This mechanism may be the key reason for indatraline to cause inhibition of cell proliferation in HeLa and SMCs.

Our results suggest the possibility that autophagy induction could be mediated by its known target, the monoamine transporters, or by another target protein that may be related to the AMPK/mTOR/S6K signaling axis. Nevertheless, the identification of indatraline as a novel inducer of autophagy may not only serve as a new lead for developing new therapeutic agents for restenosis, it may additionally serve as a novel molecular probe for studying the regulation of autophagy.

### Experimental Procedures

#### Cell culture and transfection

A stable COS7 cell line expressing EGFP-LC3 was established by transfecting COS7 cells with pEGFP-LC3 using Lipofectamine 2000 according to the manufacturer’s protocol (Invitrogen, CA, USA). Stable clones were selected in complete medium containing 750 μg/mL of G418. Human cervical adenocarcinoma (HeLa) cells were obtained from the Korean Cell Line Bank (Seoul, South Korea) and were maintained in Dulbecco’s Modified Eagle Medium (DMEM) containing 10% fetal bovine serum (FBS). Human smooth muscle cells (SMCs) were purchased from Lonza and maintained in SmBm medium (Lonza, MD, USA). Cells were cultured at 37 °C in an atmosphere of 5% CO_2_ in air, pH 7.4. DMEM and FBS were obtained from Gibco Laboratories. Plasmids encoding EGFP-LC3 and the anti-LC3 antibody were kindly provided by Tamotsu Yoshimori (National Institute for Basic Biology, Okazaki, Japan).

For target gene knockdown, cells were transfected with siRNA using Lipofectamine RNAiMAX (Invitrogen) according to the manufacturer’s protocol; siRNAs for ATG5 and ATG7 were purchased from Dharmacon: ON-TARGET plus SMARTpool Human ATG7 (Cat. No. L-020112-00-0005) and ATG5 (L-004374-00-0005). Starvation was induced during the transfection, and serum (10% FBS) was added with drug treatment.

### Drug library

The JHDL was prepared with 10 mM stock solutions with DMSO for each compound^22^. The stock solutions were arrayed in 32 plates (96-well). Each solution in these master plates was diluted with PBS to make 200 μM stock plates used for cell treatment, and these stock plates were stored at −20 °C.

### Autophagy detection with LysoTracker, monodansylcadaverine (MDC), and the EGFP-LC3 stable cell line

COS7 cells stably expressing EGFP-LC3 were treated with compounds for 24 h and then visualized by fluorescence microscopy (IX71, Olympus, Tokyo, Japan). Fluorescence intensity was quantitatively measured with Image J 1.43u and expressed as arbitrary units. HeLa cells were cultured up to 70% confluence and then treated with indatraline for 24 h followed by treatment with Hoechst 33258 (50 nM Molecular Probes, OR, USA), LysoTracker Red (50 μM Molecular Probes), and MDC (50 μM Biochemika, Buchs, Switzerland) for 30 min. Each sample was washed with PBS three times and visualized by fluorescence microscopy.

### High-content screening (HCS)

HCS (Thermo Scientific, MA, USA) was used to quantitatively screen autophagy inducers. HeLa cells were plated in 96-well plates (5000 cells/well) and cultured for 24 h. Compounds were added to a final concentration of 10 μM for 24 h. Autophagic vacuoles were stained with LysoTracker Red. Plates were washed with PBS three times and analyzed by Cellomics^®^ ArrayScan^®^ VTI (Thermo Scientific). The Target Activation protocol approved by Thermo Scientific High-Content Informatics (HCI) was used to measure the fluorescence intensities of the nuclear and cytosolic fractions.

### Transmission electron microscopy (TEM)

Cells were harvested, washed twice with PBS and fixed with 2% paraformaldehyde/2% glutaraldehyde/0.5% CaCl_2_ (pH 7.4) for more than 6 h; subsequently, they were washed in 0.1 M phosphate buffer, which was followed by 1% OsO_4_ (in 0.1 M PBS) fixation for 2 h. After fixation, cells were exposed to 95% alcohol for dehydration and kept in propylene oxide for 10 min. Cells were incubated in a 1:1 solution of EPON mixture (EPON 812, MNA, DDSA, DMP30) and propylene oxide overnight and embedded. Ultrathin sections were prepared using an LKB 8800 Ultratome III and observed with a JEM-1011 JEOL transmission electron microscope.

### Immunoblotting

Soluble proteins were harvested from indatraline-treated HeLa cells by using sodium dodecyl sulfate (SDS) lysis buffer (50 mM Tris HCl, pH 6.8, containing 10% glycerol, 2% SDS, 10 mM dithiothreitol, and 0.005% bromophenol blue). Equal volumes of proteins were separated by 8% or 12.5% SDS-PAGE and transferred to polyvinylidene fluoride membranes (Millipore). Blots were then blocked and immunolabeled overnight at 4 °C with primary antibodies, LC3 (MBL), p-mTOR, mTOR, p-AKT, AKT, p-S6K, S6K (Cell Signaling), and tubulin (Upstate Biotechnology). Immunolabeling was visualized using an enhanced chemiluminescence kit (Amersham Life Science, Inc.) according to the manufacturer’s instructions. Tubulin was used as an internal control.

### Immunofluorescence

HeLa cells were fixed with 4% formaldehyde for 5 min and washed with PBS. Cells were permeabilized with 0.1% Triton-X 100 for 5 min and washed with PBS. Bovine serum albumin (BSA, 1%) was used as blocking buffer and blocking was done for 30 min. Primary antibody was treated for 1 h and washed with PBS. Secondary antibody and Hoechst 33342 was treated for 1 h and washed with PBS. Images were analyzed from Carl Zeiss LSM 510 META confocal microscope.

### Cell proliferation and viability assay

Cell proliferation was measured using an MTT [3-(4,5-dimethylthiazol-2-yl)-2,5-diphenyltetrazolium bromide] colorimetric assay. Cells were seeded at a density of 3 × 10^3^ cells/well in 96-well plates and incubated for 24 h. Cells were treated with indatraline at various concentrations. Three days after treatment, 2 mg/mL MTT was added to each well and incubated for 4 h. MTT formazan in each well was dissolved in 150 μL of DMSO, and the absorbance at 595 nm was measured using a microplate reader (BioTek Instruments Inc., VT, USA). Relative cell growth was measured by calculating the ratio between the signal of indatraline-treated wells and that of control wells. Cell viability was examined by Trypan Blue staining. Seeding was performed with 10^4^ cells/mL 24-well plates, and the cells treated with the drug for designated times. Cells were harvested by trypsinization and stained with Trypan Blue Stain (Gibco, USA). The numbers of live and dead cells were counted, and the ratio of live cells to total cells was calculated.

### ATP activity monitoring

Three thousand cells were seeded onto the white 96-well plates, and ATP production level of HeLa cells upon indatraline treatment was measured using the ATPlite 1step Luminescence Assay System as directed by the manufacturer’s protocol (Perkinelmer Life Sciences, Boston, MA).

### Rat restenosis model

Animal studies were approved and performed in accordance with the guidelines of Institutional Animal Care and Use Committee (IACUC) of Ewha Womans University and conformed to *Guide for Care and Use of Laboratory Animals* published by the US National Institutes of Health (The National Academies Press, 8^th^ Edition, 2011). The ten-week-old male Sprague-Dawley rats (Charles River, U.S.A.) were used for a balloon-induced injury model. A balloon injury was created with an infiltrated 2F Fogarty balloon catheter in the normal left rat carotid artery. Ten-week-old male rats were anesthetized, the left external carotid artery was exposed, and its branches were electrocoagulated. A catheter was pushed 1 cm through the transverse arteriotomy of the external carotid artery, and endothelial denudation was achieved by three passes along the common carotid artery. After balloon injury, indatraline, rapamycin, or DMSO was injected into the injured carotid arterial region through a catheter and incubated for 15 min. At 1 week or 10 days after injury, the common carotid arteries were excised after transcardiac perfusion-fixation with heparinized saline containing 3.7% formaldehyde and then were paraffin-embedded. Five serial tissue sections (100-μm interval and 3-μm thickness) were obtained from the middle area of the common carotid arteries. Each slide was stained with hematoxylin and eosin (H&E).

### TUNEL assay

The paraffin sections were incubated in PBS containing 0.1% Triton X-100 for 10 min. Then, Terminal deoxynucleotidyl transferase (TdT) dUTP Nick-End Labeling (TUNEL) reactions were performed for 1 h at 37 °C using the *In Situ* Cell Death Detection Kit, Fluorescein (Roche Diagnostics Corp., Mannheim, Germany), according to the manufacturer’s instructions. Cell nuclei were counterstained with 4′,6-diamidino-2-phenylindole (DAPI).

### Morphometric analysis

Four different areas (lumen, intima, media, and total vascular area) were measured in sections stained with H&E using analyzing software (NIH Image 1.62; http://rsb.info.nih.gov/nih-image/download.html). The areas surrounded by the luminal surface, internal elastic lamina (IEL), and external elastic lamina (EEL) were measured. The intimal area was determined by subtracting the luminal area from the area defined by the IEL, and the medial area was calculated by subtracting the area defined by the IEL from the area defined by the EEL. Five sections were measured in each animal.

## Additional Information

**How to cite this article**: Cho, Y. S. *et al.* Antidepressant indatraline induces autophagy and inhibits restenosis via suppression of mTOR/S6 kinase signaling pathway. *Sci. Rep.* **6**, 34655; doi: 10.1038/srep34655 (2016).

## Supplementary Material

Supplementary Information
