# Supplementary material for: Antidepressant indatraline induces autophagy and inhibits restenosis via suppression of mTOR/S6 kinase signaling pathway
Source: Sci Rep. 2016 Oct 3;6:34655. doi: 10.1038/srep34655 (PMC5046148; doi:10.1038/srep34655)
Supplement: Supplementary Information [file srep34655-s1.pdf]

Supplementary Information

Antidepressant indatraline induces autophagy and inhibits restenosis via suppression of mTOR/S6 kinase signaling pathway

Yoon Sun Cho[1], Chih-na Yen[1,] Joong Sup Shim[3], Dong Hoon Kang[4], Sang Won Kang[4], Jun O. Liu[3], and Ho Jeong Kwon[1,2*]

[1]Chemical Genomics Global Research Laboratory, Department of Biotechnology, Translational Research Center for Protein Function Control, College of Life Science & Biotechnology, Yonsei University, Seoul 120-749, Republic of Korea

[2]Department of Internal Medicine, Yonsei University College of Medicine, Seoul 120-752, Republic of Korea.

[3]Department of Pharmacology and Molecular Sciences, Johns Hopkins School of Medicine, 725 N. Wolfe St, Baltimore, MD 21205

[4]Division of Life and Pharmaceutical Sciences, Ewha Womans University, Seoul 120-750, Republic of Korea

*Correspondence should be addressed to Ho Jeong Kwon, [1]Chemical Genomics Global Research Laboratory, Department of Biotechnology, Translational Research Center for Protein Function Control, College of Life Science & Biotechnology, Yonsei University, Seoul 120-749, Republic of Korea, Tel:82-2-2123-5883; Fax:82-2-362-7265; E-mail: kwonhj@yonsei.ac.kr

Supplementary Figure 1

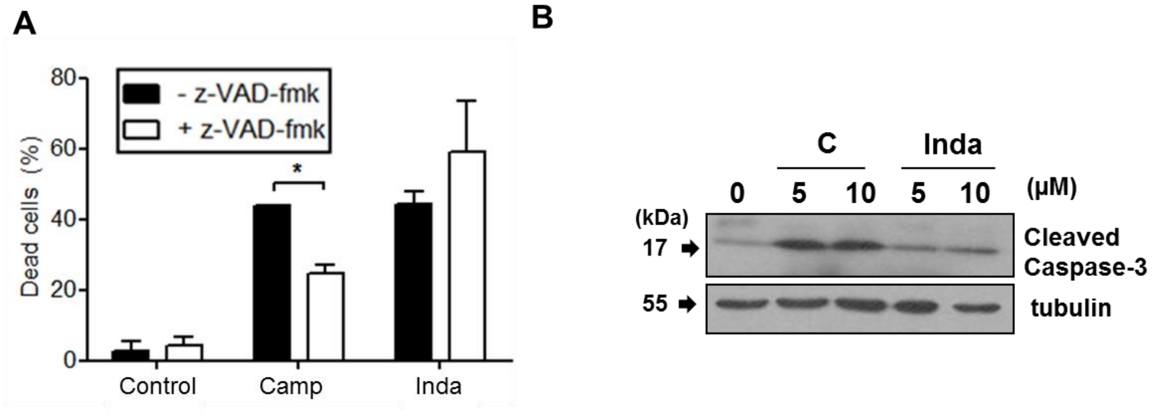

Supplementary Figure 1. Indatraline inhibits cell growth and induces apoptosis-independent cell death. (A) Effect of caspase inhibitor, z-VAD-fmk (10 μM) on indatraline-induced cell death (NT: control DMSO, Indatraline 10 μM, camptothecin 10 μM). (B) Cleaved caspase-3 levels in indatraline-treated cells (C: camptothecin, I: indatraline).

Supplementary Figure 2

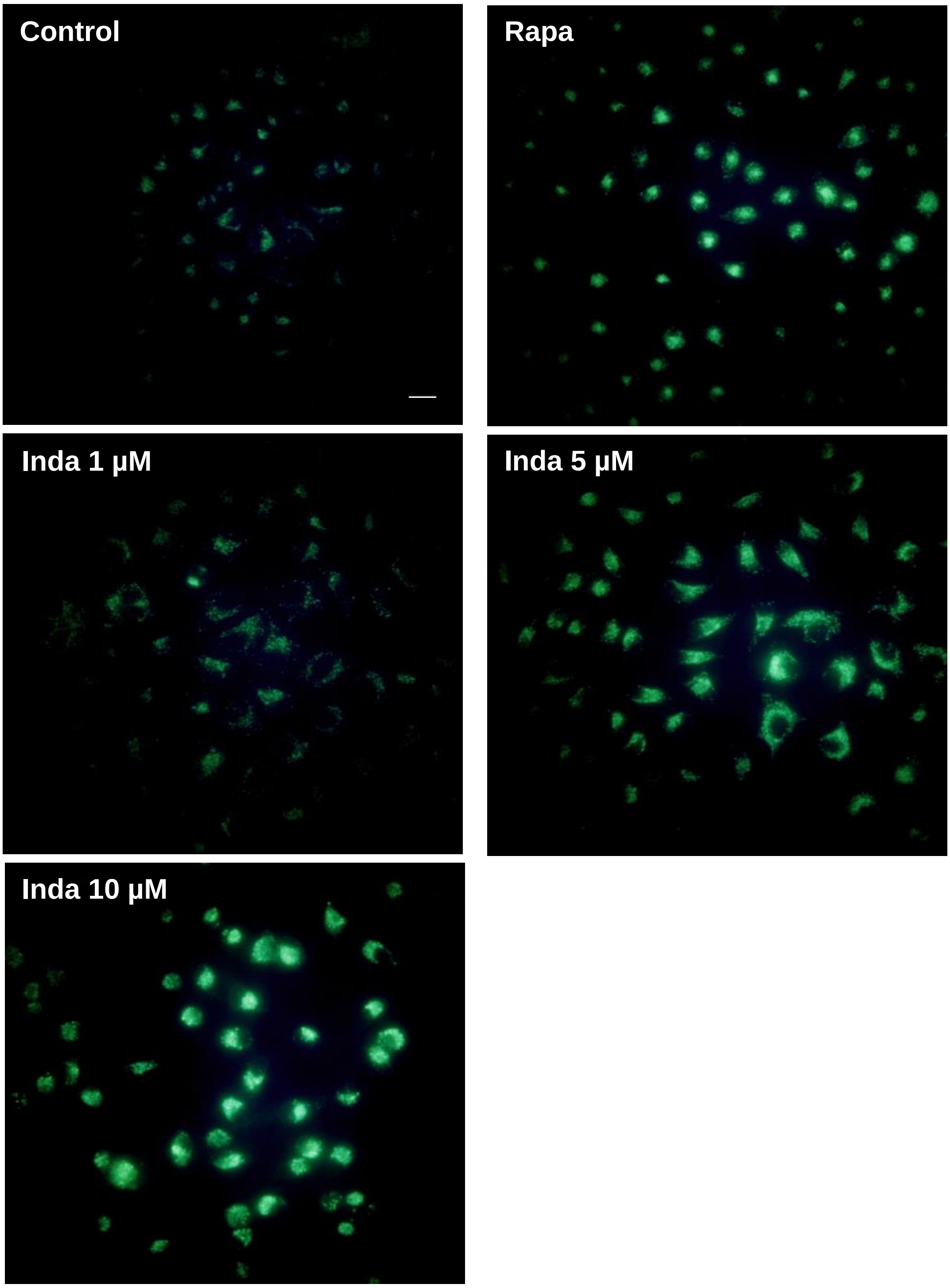

Supplementary Figure 2. Microscopy fluorescence images of MDC in Inda and rapamycin treated cells after 24 hr. Representative images from three independent experiments.

Supplementary Figure 3A

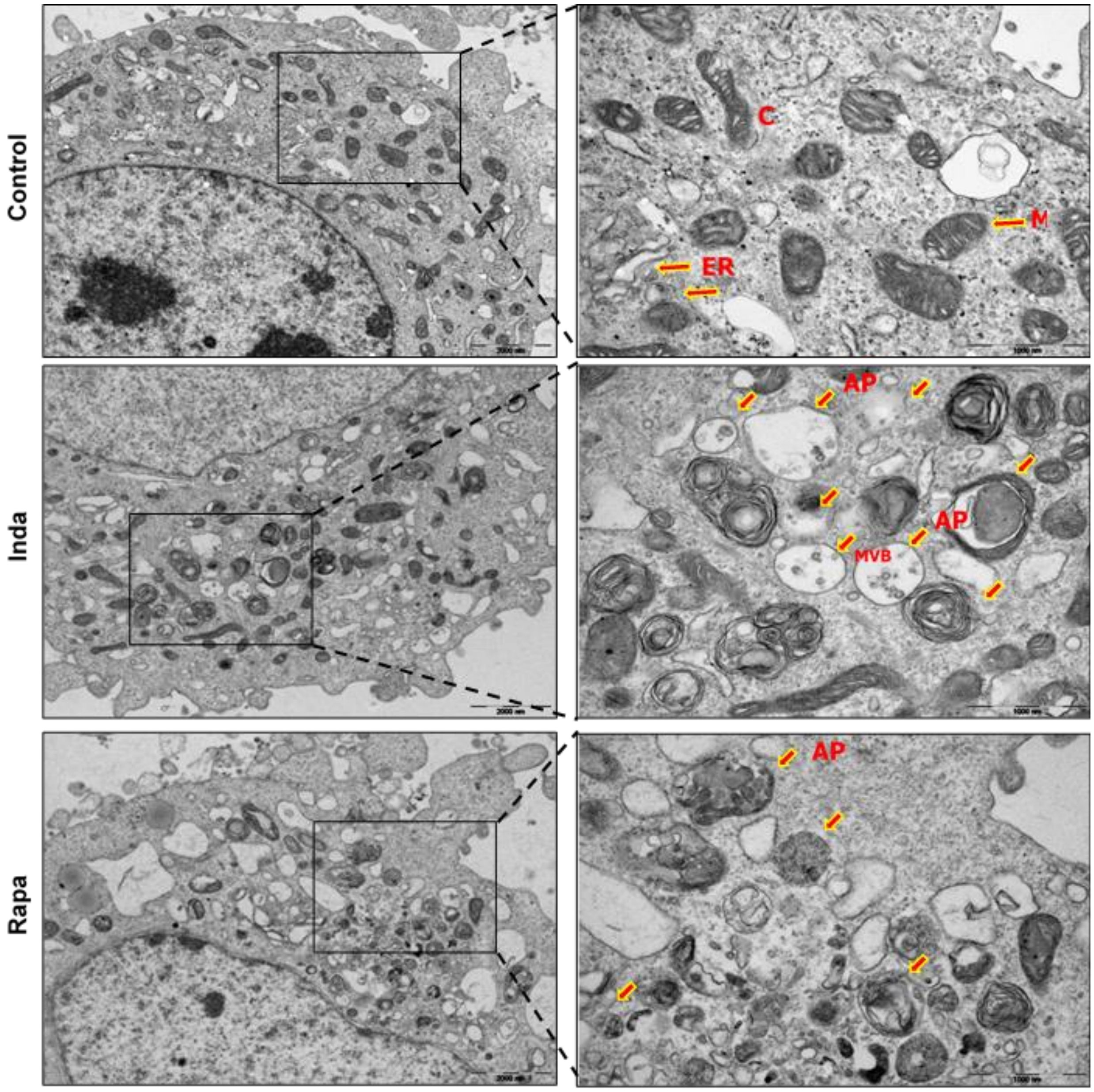

Supplementary Figure 3B

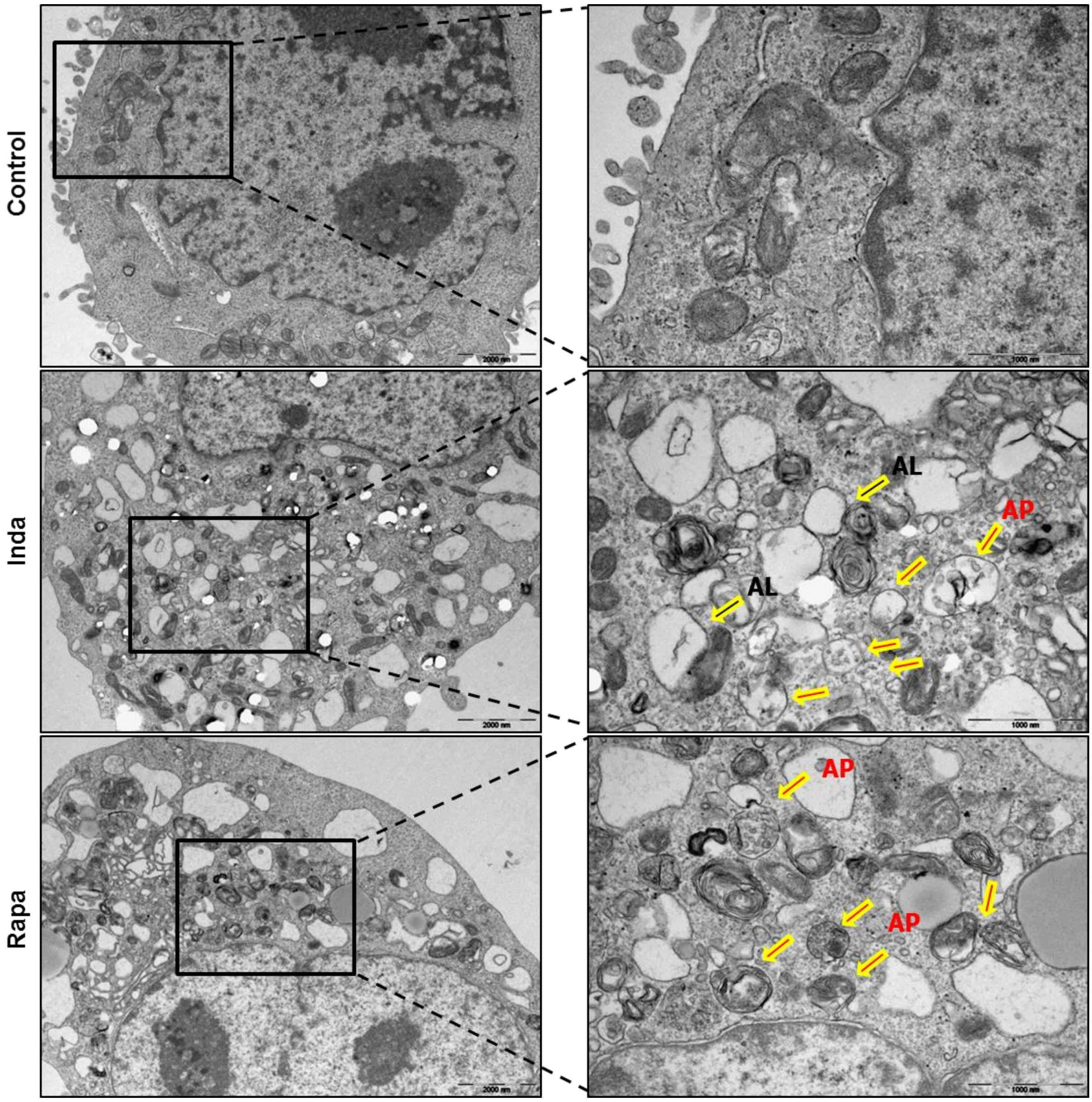

Supplementary Figure 3. TEM images of autophagic vacuoles after 12 hr (**3A**) and 24 hr (**3B**) of drug treatment in HeLa cells. The arrows labeled represent the following: AL: autophagolysosome, AP: autophagosome, MVB: multi-vesicular bodies, M: mitochondria, C: cytosol, and ER: endoplasmic reticulum. Scale bars represent 2000 nm and 10000nm.

Supplementary figure 4

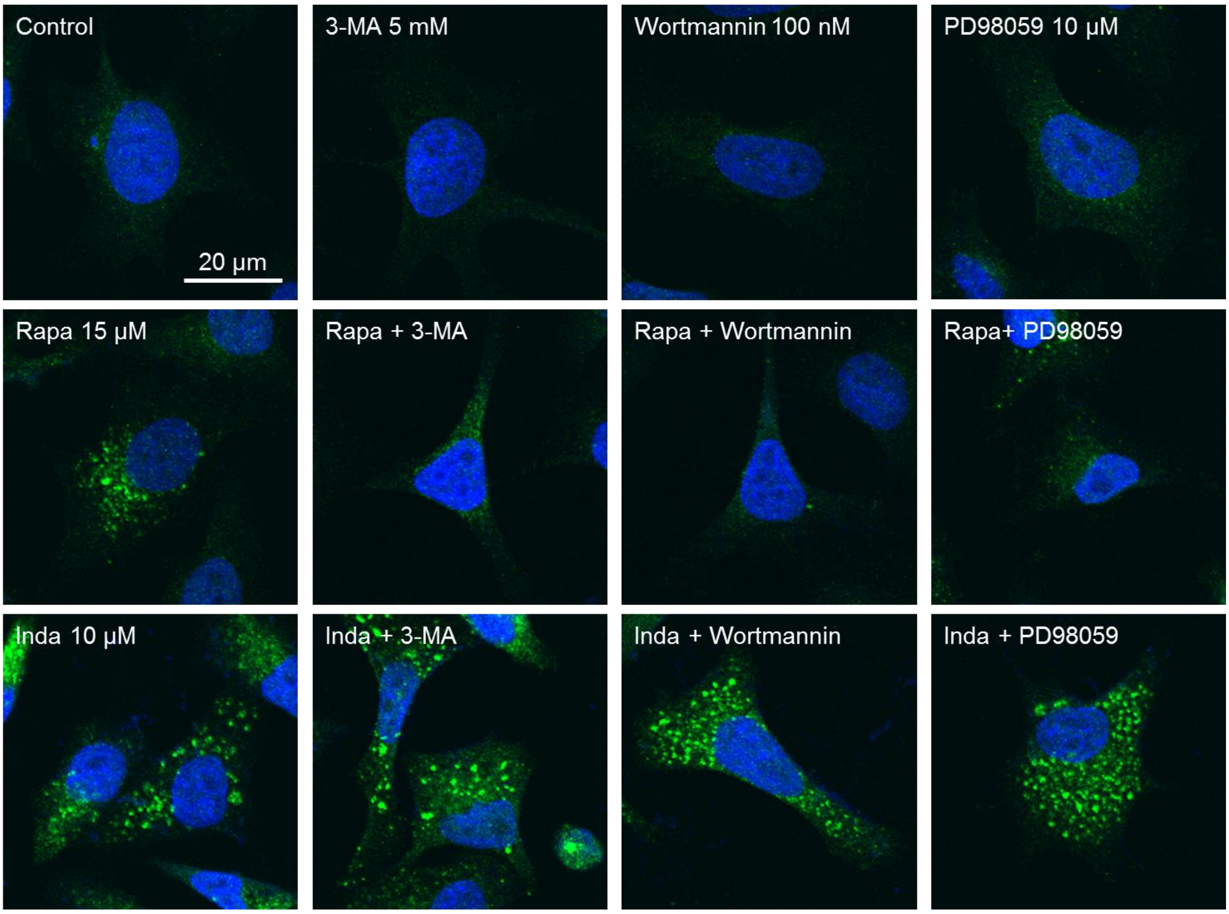

Supplementary figure 4. Immunoblotting of LC3 in drug treated HeLa cells after 24 hr. 3MA, Wortmannin, PD98059 were treated 1 hr before indatraline or rapamycin treatment.
